# Supporting Adolescents With HIV in South Africa Through an Adherence-Supporting App: Mixed Methods Beta-Testing Study

**DOI:** 10.2196/47575

**Published:** 2023-06-01

**Authors:** Marta I Mulawa, Bulelwa Mtukushe, Elizabeth T Knippler, Mluleki Matiwane, Maryam Al-Mujtaba, Kathryn E Muessig, Jacqueline Hoare, Lisa B Hightow-Weidman

**Affiliations:** 1 School of Nursing Duke University Durham, NC United States; 2 Duke Global Health Institute Duke University Durham, NC United States; 3 Department of Psychiatry and Mental Health University of Cape Town Cape Town South Africa; 4 Department of Health Behavior Gillings School of Global Public Health The University of North Carolina at Chapel Hill Chapel Hill, NC United States; 5 Institute on Digital Health and Innovation College of Nursing Florida State University Tallahassee, FL United States; 6 Division of Infectious Diseases School of Medicine The University of North Carolina at Chapel Hill Chapel Hill, NC United States

**Keywords:** adolescents, HIV, adherence, mHealth, mobile health, technology, smartphone app, health app, beta testing, implementation, digital health intervention, usability, HIV treatment

## Abstract

**Background:**

Novel smartphone app–delivered interventions have the potential to improve HIV treatment adherence among adolescents with HIV, although such interventions are limited. Our team has developed *Masakhane Siphucule Impilo Yethu* (MASI; Xhosa for “Let's empower each other and improve our health”), a smartphone app–delivered intervention to improve treatment adherence among adolescents with HIV in South Africa. MASI was adapted to the South African cultural context using the HealthMpowerment platform, an evidence-based digital health intervention developed for and with youth in the United States.

**Objective:**

We conducted this beta-testing study to (1) explore the initial usability of MASI, (2) examine engagement and experiences using MASI features, and (3) inform refinements to the app and intervention implementation plan prior to a subsequent pilot randomized controlled trial (RCT).

**Methods:**

This study was conducted from August 2021 to December 2021 in Cape Town, South Africa. Beta-testing participants received access to MASI for 3 weeks. A mixed methods approach was used, with brief questionnaires and semistructured in-depth interviews conducted prior to app installation and after 1 week to 2 weeks of app testing. Engagement with MASI was measured through analysis of back-end app paradata, and follow-up in-depth interview guides were tailored to each participant based on their app use.

**Results:**

Participants in the beta-testing study (6 male participants, 6 female participants; ages 16-19 years) collectively spent 4.3 hours in MASI, averaging 21.4 minutes per participant over the 3-week period (range 1-51.8 minutes). Participants logged into MASI an average of 24.1 (range 10-75) times during the study period. The mean System Usability Scale score was 69.5 (SD 18), which is considered slightly above average for digital health apps. Thematic analysis of qualitative results revealed generally positive experiences across MASI features, although opportunities to refine the app and intervention delivery were identified.

**Conclusions:**

Initial usability of MASI was high, and participants described having a generally positive experience across MASI features. Systematically analyzing paradata and using the interview findings to explore participant experiences allowed us to gain richer insights into patterns of participant engagement, enabling our team to further enhance MASI. The results from this study led to a few technological refinements to improve the user experience. Enhancements were also made to the intervention implementation plan in preparation for a pilot RCT. Lessons learned from the conduct of this beta-testing study may inform the development, implementation, and evaluation of similar app-delivered interventions in the future.

## Introduction

### Background

Adolescents living with HIV are the only age group in which HIV-related mortality is not declining [1]. Despite the recognition that adolescents face unique challenges [2,3] and regularly demonstrate poor adherence [4], interventions designed to support antiretroviral therapy (ART) adherence and care engagement among adolescents with HIV in settings like South Africa are limited [5-7]. Additionally, the findings of existing studies suggest that interventions designed to address only one specific challenge (eg, forgetfulness) are inadequate [8,9]. Such findings support the development of comprehensive interventions targeting multiple behavioral determinants, including social support, to influence ART adherence.

Novel smartphone app–delivered interventions have the potential to improve ART adherence among adolescents with HIV by comprehensively addressing their informational needs, fostering social connections among users, and presenting users with engaging activities. Although mobile phone–delivered interventions have shown promising results in improving ART adherence among adults in some sub-Saharan African settings [10-12], there exists a lack of effective app-delivered interventions for adolescents with HIV in South Africa. Furthermore, most mobile health interventions in sub-Saharan Africa are SMS text messaging–based, unlike the advanced smartphone apps developed and tested with more frequency in high-income countries [13].

Our team has developed *Masakhane Siphucule Impilo Yethu* (MASI; Xhosa for “Let's empower each other and improve our health”), a smartphone app–delivered intervention (iOS and Android) to improve treatment adherence among adolescents with HIV in South Africa. MASI features include a Forum moderated by peer mentors where users can interact using anonymous usernames, a Health Tracker, an Ask the Expert section, interactive Activities, and multimedia Resources. MASI was culturally adapted to the South African context using the HealthMpowerment (HMP) platform, an evidence-based digital health intervention developed for and with youth in the United States. Adaptation of HMP to the South African context was done through formative research, including in-depth interviews conducted with adolescents with HIV in this setting prior to this study [14].

### Objective

We conducted this beta-testing study to (1) explore the initial usability of MASI, (2) examine engagement and experiences using MASI features, and (3) identify refinements to the app and intervention delivery plan prior to the implementation of a subsequent pilot randomized controlled trial (RCT). The purpose of this paper is to highlight the results of the beta-testing study and share lessons learned that could inform the development and implementation of other app-delivered interventions designed to serve similar populations in the future.

## Methods

### Context

This research was conducted within a teaching hospital linked to several HIV clinics in Cape Town, South Africa. Participants for MASI beta testing were recruited from an ongoing parent study designed to improve the assessment of neurocognitive impairment among adolescents ages 15 years to 19 years with perinatally acquired HIV in the Western Cape region of South Africa. The inclusion criteria for the parent study were intentionally broad (ie, having been born with HIV, being aware of your HIV status, and having been prescribed ART) to allow recruitment of a generalizable sample of adolescents with perinatally acquired HIV who were on ART in the study area. Participants and caregivers in the parent study were regularly asked for their assent or consent to be approached about additional research studies. This information was used for recruitment into the MASI beta-testing study.

The MASI beta-testing study was conducted as formative research to inform a subsequent pilot RCT designed to assess the feasibility, acceptability, and preliminary efficacy of MASI on ART adherence and social support.

### Intervention Description

MASI is a comprehensive, mobile app–delivered intervention designed to foster connection among users and provide engaging resources related to health, life skills, relationships, and well-being. MASI uses a strengths-based approach that acknowledges and bolsters individual and social network–level resources and assets [15]. HMP, the digital health intervention platform upon which MASI was built, was developed and extensively tested with youth in the United States [16]. HMP was designed to foster social support and connection among users through an interactive participant forum, provide comprehensive informational resources, and present users with engaging activities [17]. An earlier version of HMP resulted in significant reductions in risky sexual behaviors when tested with young Black/African American men who have sex with men [18] and was designated an Evidence-Informed Intervention showing Good Evidence for Risk Reduction by the HIV/AIDS Prevention Research Synthesis project at the Centers for Disease Control and Prevention [19]. The latest version of HMP, designed to address intersectional stigma among Black and Latinx men who have sex with men and transgender women, is being evaluated in an RCT in the United States [17]. The HMP platform has also been adapted for use in over 10 projects currently underway or in development in a variety of settings [20-26].

**Figure 1:**
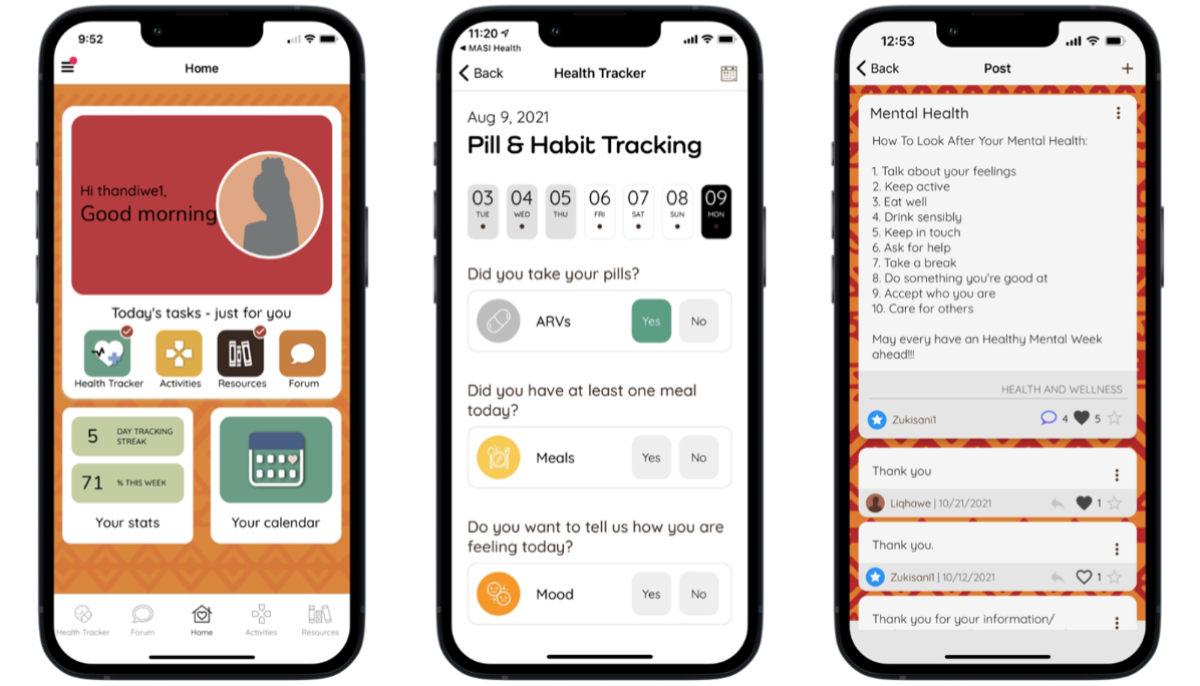
Screenshots of the Masakhane Siphucule Impilo Yethu (MASI) home page, Health Tracker, and Forum features.

**Figure 2:**
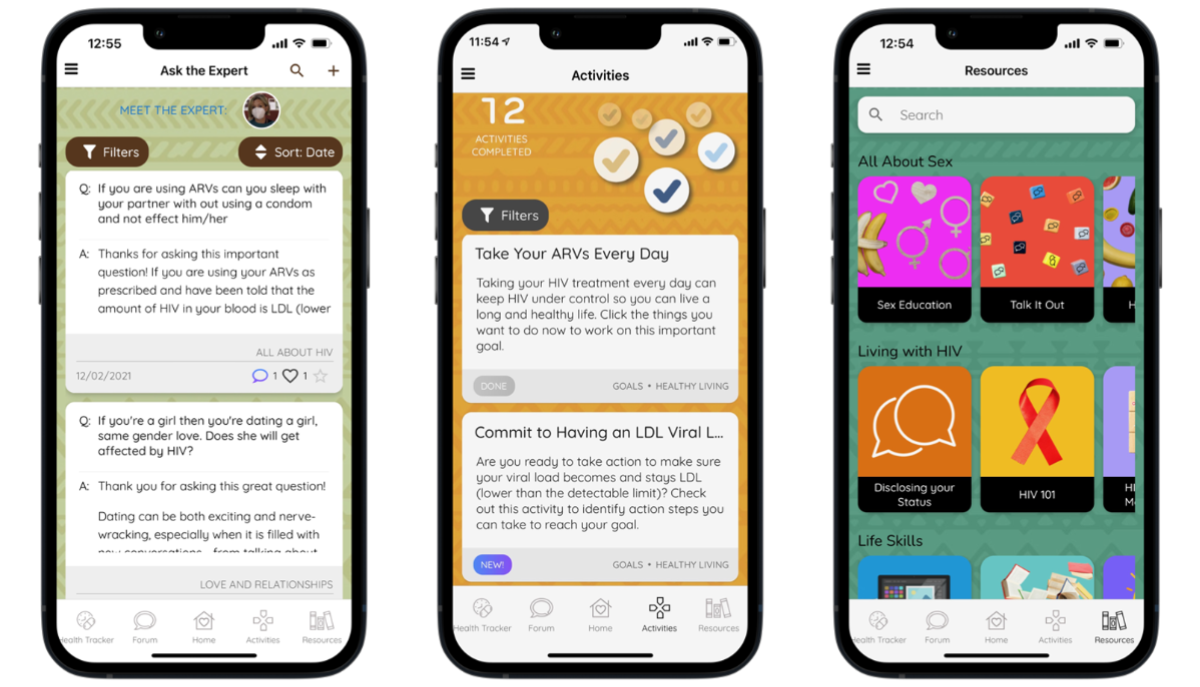
Screenshots of the Masakhane Siphucule Impilo Yethu (MASI) Ask the Expert, Activities, and Resources features.

MASI includes a range of features including a Health Tracker, a Forum moderated by peer mentors where users can interact using anonymous usernames (Figure 1), an Ask the Expert section, interactive Activities, and a multimedia Resources feature (Figure 2). The Health Tracker guides users to schedule customizable reminders, track their adherence and other health behaviors, and view graphs and visuals of their tracked data. The Forum provides a platform for participants to interact with other MASI users and peer mentors by starting or adding to existing discussion posts containing text, images, video, or links. The Ask the Expert feature allows users to privately submit questions that are then answered by a health professional and posted anonymously. Both the Forum and Ask the Expert features were prepopulated with posts and questions developed by the research team to serve as examples and encourage user-generated contributions. The Activities section includes a variety of activity types (eg, quizzes, goal setting, decision aids) covering a broad range of health-related topics including living with HIV, love and relationship, sexual health topics, and life skills; the Resources feature includes multimedia resources and information on similar topics.

MASI’s home page includes a list of “Today’s tasks” and buttons for 4 app features: Health Tracker, Activities, Resources, and Forum. The Activities and Resources buttons take participants to a specified activity or resource for the day. Once completed, the home screen displays a red check mark on the feature’s button in the “Today’s tasks” section; during the beta-testing study, if participants clicked on the button again, they received a congratulatory message that, if clicked again, brought them to the main landing page for that feature. The Health Tracker button on the home screen brings the user to a screen where they can record their pills and other selected health stats for that day. The Forum button under “Today’s tasks” is another way for participants to access the Forum feature. MASI also automatically collects detailed intervention usage metrics (ie, paradata) [27,28] on where and how each user accesses and moves through the intervention and uses its components.

### Participants

Adolescents with HIV were eligible to participate if they were enrolled in the ongoing parent study and reported having daily access to an Android or iOS smartphone meeting the minimal requirements (operating system: Android version 5.0 or higher or iOS version 10 or higher; phone having at least 200 MB in available storage to accommodate download of the intervention app). Participants were sampled to maximize variation regarding age and gender.

### Procedures

This study was conducted from August 2021 to December 2021 in Cape Town, South Africa. Participants were recruited and screened by phone and, if eligible and interested, were invited for a study enrollment visit. Participants were transported to study visits using a registered transport company to reduce transportation barriers and last-minute cancellations. After completing the informed consent or assent process (including consent from an adult caregiver for participants under 18 years old), participants completed a brief interviewer-administered survey about their demographics, smartphone specifications and use, and adherence to HIV treatment. Survey data were then double entered into REDCap, a secure, web-based application hosted at Duke University.

The first in-depth interview focused on the adolescents’ opinions about the look and feel of the app and study logo and on earning points and badges used in the app. During the initial in-depth interview, we installed MASI on each participant’s phone. MASI was accessed through the Google Play Store or App Store. After the participants created a unique username, password, and PIN, we demonstrated how to use each feature of the app. As participants were shown how to use the app, we asked questions to obtain their feedback on the app features and the process of setting up MASI.

Participants were asked to set daily reminders for tracking ART adherence in the Health Tracker and to use MASI daily to track their adherence while exploring other features. Participants were also informed that we would be able to know what they did in the app and how much time they spent in the various features. Participants were provided 1 GB of smartphone data to support their use of the app during the 3-week study.

Follow-up study visits were conducted 1 week to 2 weeks after the enrollment visit. At this visit, another brief interviewer-administered survey was conducted to assess MASI usability and treatment adherence. Usability was measured with the 10-item System Usability Scale (SUS), in which total scores range from 0 to 100 and scores >68 are considered “above average” [29,30]. Follow-up data were entered directly into the REDCap data collection software by the interviewers. The follow-up in-depth interview focused on participants’ experiences using MASI, opinions about information in the app, potential app improvements, and feelings about support received through the app.

Prior to the follow-up interview, we analyzed the participants’ backend paradata from the previous 1 week to 2 weeks to create a user-specific engagement summary. Based on the summary, we customized the follow-up interview guide to probe about their unique experience with different features. For example, 1 participant who logged in for 6 days and then stopped logging in was asked, “I can see you stopped using MASI after November 21st. Can you help me understand why your use of MASI changed around this time?” Participants who used the Ask the Expert feature were asked about their motivation to post a question and how they felt about the response; those who did not ask a question were probed about their reasons for not using the feature. Participants were also asked whether they would recommend any changes be made to app features, had encountered any technical difficulties, or had any privacy or confidentiality concerns. Participants received R250 (US $14.75) after each in-depth interview as compensation for their time.

### Ethics Approval

The study was approved by the University of Cape Town Faculty of Health Sciences Human Research Ethics Committee (HREC REF: 606/2020) and the Duke University Health System Institutional Review Board (Protocol ID: Pro00103309).

### Data Analysis

MASI paradata were extracted from the app’s backend SQL database and analyzed in R. To explore engagement with MASI features that allowed the posting of user-generated content (ie, Forum and Ask the Expert), we categorized participants based on their level of engagement (eg, posting, commenting or replying, liking or favoriting, or reading content). The brief survey data were summarized using descriptive statistics (means, SDs, ranges, percentages, and frequencies) in R.

The in-depth interviews were audio recorded. For each interview, the interviewers generated detailed summaries describing findings related to each topic discussed. The summaries were shared with the research team immediately after the interview to inform subsequent interviews and ongoing analyses. The follow-up in-depth interviews were then simultaneously translated and transcribed into English by a bilingual translator or transcriptionist. Translations and transcriptions were not obtained for the initial in-depth interviews, which focused mostly on brief feedback about the app’s logo and badges and primarily comprised research staff demonstrations of how to use the app’s features and their documentation of questions raised by participants during the onboarding process. The written summaries and interview transcripts were analyzed using a rapid analysis approach [31], which focused on developing thematic summaries to facilitate comparison of findings across participants. Representative quotes were identified in team discussions and are presented along with the participant’s demographic characteristics, time spent using the app, and SUS score to contextualize the findings within the range of participant characteristics and experiences.

## Results

### Participant Characteristics

A total of 14 participants (7 female participants, 7 male participants) were enrolled in the study and completed the brief survey; 2 participants were withdrawn at enrollment because they were not able to install MASI on their devices. Characteristics of the 12 study participants who were able to install the app (6 female participants, 6 male participants) and their mobile phone devices are presented in Table 1. The mean age of study participants was 18 (range 16-19) years. All but 1 participant reported speaking Xhosa as their home language, and 9 (75%) participants were still in school.

**Table 1:** Characteristics of participants who participated in MASI beta testing (n=12).

Characteristic			Value
Age (years), mean (SD)			18 (1)
**Gender, n (%)**			
	Female	6 (50)	
	Male	6 (50)	
**Home language, n (%)**			
	Xhosa	11 (92)	
	Sotho	1 (8)	
Currently in school, n (%)			9 (75)
**Highest level of education, n (%)**			
	School of skills	1 (8)	
	Primary or less	1 (8)	
	Some secondary school	8 (67)	
	Secondary school	2 (17)	
**Smartphone operating system, n (%)**			
	Android	11 (92)	
	Apple (iOS)	1 (8)	
**Smartphone brand, n (%)**			
	Samsung	4 (33)	
	Hisense	3 (25)	
	Huawei	2 (17)	
	Apple	1 (8)	
	Mobicell	1 (8)	
	Alcatel	1 (8)	
Free storage space (GB), mean (SD)			3.1 (2.3)
**Smartphone network, n (%)**			
	MTN	5 (42)	
	Vodacom	4 (33)	
	Telkom	2 (17)	
	Cell-C	1 (8)	
Time spent using phone daily (hours), mean (SD)			8 (3)
**Frequency of app use, n (%)**			
	Every day	10 (83)	
	Most days	1 (8)	
	A few times a week	1 (8)	

### MASI Engagement

In total, participants collectively spent 4.3 hours in MASI, averaging 21.4 (range 1-50.8) minutes each. Participants logged into MASI an average of 24.1 (range 10-75) times during the 3-week period. Discrepancy between the number of log-ins and duration of time spent in MASI (eg, 1 participant logged in 10 times but spent only 1 cumulative minute in MASI) alerted the team to a systematic undercounting of time spent in the paradata. Although each log-in was consistently logged, some activities in the app were not reliably tracking the duration of time spent.

**Figure 3:**
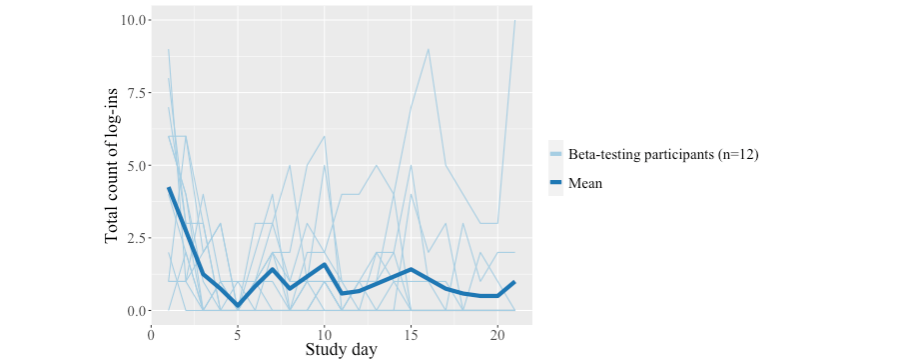
Daily count of log-ins to Masakhane Siphucule Impilo Yethu (MASI) by study day during beta testing.

An examination of log-in trends across all beta-testing participants (Figure 3) revealed that log-ins were highest in the initial days of the study. This figure also demonstrates a sustained interest in MASI over the course of the 3-week study, with daily log-ins to MASI averaging 1.2 per day.

### Overall MASI Usability

The follow-up survey and interview were completed by 11 of the 12 beta-testing participants. The mean SUS score was 69.5 (SD 18), which is considered to be slightly above average for digital health apps [30]. As part of the SUS assessment, most participants (9/11, 82%) strongly agreed or agreed that all parts of the MASI app worked well together, and all but 1 (10/11, 91%) endorsed feeling very confident using MASI. The wide SD of the SUS score is largely attributed to 1 participant who strongly endorsed several critical items including that MASI was “complicated to use” and feeling as though he would need to “learn a lot of things” before using MASI*.*

Most participants strongly agreed or agreed with additional supplemental usability and acceptability items (Table 2). All participants strongly agreed or agreed that, overall, they were satisfied with the app, and most (10/11, 91%) reported that they trusted the information in MASI and felt that the information was easy to understand. All participants strongly agreed or agreed that they would recommend the MASI app to a friend who was also using ARVs (antiretrovirals, the term adolescents with HIV typically use to refer to their ART in this setting).

During follow-up interviews, most participants reported having positive experiences with MASI, with several participants describing it as “very easy” and reporting no problems or slowdowns. One participant noted the helpful visuals within MASI that promoted ease of navigation:

[Using MASI] was not at all difficult as everything is shown very clearly. There are icons on the home screen that show you where to go. So, I did not find it difficult, I did not have to think when I needed to navigate the app. It shows everything that you want.

16-year-old female participant; time spent=11.3 minutes; SUS score=70

Several participants noted that they were unable to use MASI at times because of cellular network challenges or load-shedding in their area that turned off the electricity for some time. When prompted about challenges using the app, most participants reported no challenges. However, 1 participant found it difficult to navigate the app and understand the content or purpose of the app features:

I did not know how to navigate the app. I even tried to check the Activities, but I could not focus or understand it well, so I had to leave the Activities. I went to the Health Tracker, but I did not know how to use it too.

17-year-old male participant; time spent=54.5 seconds; SUS score=20

A few participants described technical aspects of the app that limited their ability to navigate smoothly through different features while using their phone for normal use. One participant described, “It was difficult for me to go back to the other feature I was working on, so I would have to log out completely from the app and log on again” (18-year-old male participant; time spent=9.3 minutes; SUS score=67.5). Another participant described having to “start from scratch, not where I stopped” after leaving MASI to check messages on WhatsApp, causing the app to lag (17-year-old female participant; time spent=18.5 minutes; SUS score=80).

**Table 2:** Strong agreement or agreement with supplemental usability and acceptability items (n=11).

Characteristic	Responses, n (%)
Overall, I am satisfied with the MASI^a^ app.	11 (100)
I would recommend the MASI app to a friend who was also using ARVs^b^.	11 (100)
I like the look and style of the MASI app.	11 (100)
I enjoy using the MASI app.	10 (91)
I trust the information in the MASI app.	10 (91)
The information in the MASI app is easy to understand.	10 (91)
The MASI app helps me make healthier choices.	10 (91)
The MASI app met my ARV adherence needs.	10 (91)
The MASI app helped me deal with challenges taking my ARVs.	10 (91)
The MASI app helped me to take my ARVs.	9 (82)
I understand how to use all the features in the MASI app.	8 (73)

^a^MASI: Masakhane Siphucule Impilo Yethu.

^b^ARVs: antiretrovirals.

### Engagement Across MASI Features

Among all MASI features, beta-testing participants spent the most time in the Health Tracker, followed by the Forum, Resources, Activities, and Ask the Expert.

#### Health Tracker

In total, beta-testing participants collectively spent 1 hour in the MASI Health Tracker. Several participants commented that the medication adherence tracking feature worked as intended; as 1 participant noted, “[It] reminded [me] at the right time” (19-year-old male participant; time spent=30.1 minutes; SUS score=67.5). One participant noted that, as a result, she “stopped forgetting taking [her] medication” (16-year-old female participant; time spent=11.3 minutes; SUS score=70).

Several participants appreciated that the Health Tracker allowed them to track other behaviors; they particularly appreciated its ability to track meals and mood in addition to their ART adherence:

I would say it helped me remember my meals, because sometimes I really forget to eat. So, sometimes, nobody really asks me how I am doing and stuff with my mood, so it does help because I'm not the person who likes food, so that is why I chose to track my meals.

19-year-old male participant; time spent=34.8 minutes; SUS score=85

#### Forum

The next most-used feature (in terms of time spent) was the participant Forum. Beta-testing participants collectively spent 47.5 minutes in the MASI Forum. In follow-up interviews, participants noted that they “liked the Forum” and felt that the “welcome by peer mentors was nice” (17-year-old female participant; time spent=18.5 minutes; SUS score=80). When reflecting on what was likeable about the Forum, another participant noted that he appreciated that the Forum helped him feel connected to others:

Knowing that you are not alone here, there's also other people who are living with HIV. So, whenever I saw the posts, I would feel like someone [was] saying something that is not only about him or her, but...involves other people.

19-year-old male participant; time spent=30.1 minutes; SUS score=67.5

At least one user-generated main post in the Forum was contributed by 2 participants, and 4 additional participants posted at least one comment or reply. An additional 3 participants did not contribute any user-generated content but clicked the like button for at least one post or comment. The remaining 3 participants only viewed content posted by others.

Most posts and comments in the Forum were in response to introductory posts and motivational or informational posts by the MASI peer mentors. However, 1 beta-testing participant contributed a user-generated post related to suicidal ideation, asking “Have you ever tried killing yourself because you are living with HIV?” (18-year-old male participant; time spent=9.3 minutes; SUS score=67.5). The admin team responded with a reply to the Forum post (visible to all participants) that linked to local mental health resources available to participants (this response was later incorporated as example text into a protocol to establish standardized procedures in response to user-generated content of concern). Additionally, both peer mentors posted responses in which they shared personal experiences and feelings as well as motivation for the participant; the participant later noted how appreciated and helpful the peer mentors’ responses had been (“it was quite motivating”). This feedback underscored the importance of having peer mentors engage actively in the MASI app, respond to user-generated content, and relate to participants as fellow young people living with HIV.

The relatively small amount of user-generated content in the Forum was confusing to at least one participant, who described feeling concerned when he did not see many posts from other people. Having anticipated that there would be more activity in the Forum, the participant described feeling “quite stressed...because I felt like I was being left behind” (17-year-old male participant; time spent=54.5 seconds; SUS score=20).

When participants who had not posted nor commented in the Forum were asked during their follow-up in-depth interviews why they chose not to contribute, they reflected on barriers to their active engagement. One participant, who had accessed the Forum 20 times over 10 days of MASI use, described feeling too shy to post before others did and proposed adding ice breakers or “questions directed to adolescents” to “encourage me to continue using the app” and “get other people’s views as well” (19-year-old male participant; time spent=12.7 minutes; SUS score=75). Similarly, another participant expressed interest in being given specific topics to discuss. Other participants with low levels of active engagement indicated that they were unsure of what to post, did not feel they had anything to say, or were hesitant to post as there were so few posts in the Forum.

Notably, when asked about what could be done to improve the Forum, several participants mentioned that they were satisfied with the Forum in its current form; they simply were not using the feature because they preferred other features to the Forum:

I wasn’t very much interested on the Forum...what was more interesting to me was the articles...It is not that there is something I dislike about the Forum. I like the Forum. But I did not feel like spending time on the Forum.

16-year-old female participant; time spent=11.3 minutes; SUS score=70

#### Resources

Beta-testing participants collectively spent 44.4 minutes in the MASI Resources feature and opened a total of 93 articles. The 12 participants opened 7.8 (range 0-29) articles on average during the study period. Participants accessed the Resources feature on an average of 5.4 (range 0-19) days. The paradata revealed that 62% (57/93) of the articles opened were accessed by participants who clicked on the Resources button in the “Today’s tasks” section of the home page. In addition, we observed that many participants apparently accessed only 1 resource each day; on over 75% (49/65) of the days on which an article was opened by a participant, the participant opened only 1 article. When this trend was explored in a follow-up in-depth interview with 1 participant, she described how the pop-up message on the home page had influenced the number of resources and activities she viewed each day:

I would say [I completed] one a day because whenever I would go to the app, it would tell me that the activity is closed. Which shows that I have done the activity.

17-year-old female participant; time spent=39.6 minutes; SUS score=72.5

These data suggested that the home page was an important mechanism for alerting participants to MASI resources and that the check mark and corresponding completion message may have influenced participants to stop for the day rather than encouraging them to use the feature buttons at the bottom of the screen to continue exploring additional resources.

Additionally, when monitoring engagement to prepare the user-specific engagement summary, we observed that 1 participant was searching the Resources section for terms related to same gender–loving relationships. At that time, MASI did not have articles with a specific focus on understanding gender and sexuality, or on lesbian, gay, bisexual, transgender, and queer (LGBTQ) communities. The team was able to publish relevant content while the participant was still using the platform. This insight from the paradata was particularly helpful given that the participant did not offer any recommendations or changes to the app or its content during the follow-up in-depth interview.

#### Activities

Beta-testing participants collectively spent 36.0 minutes in the MASI Activities feature and started a total of 123 activities. The 12 participants started 10.3 (range 0-34) activities on average during the study period. Participants accessed the Activities feature on an average of 5.8 (range 0-19) days. The paradata revealed that 61% (75/123) of the activities started were opened by participants who clicked on the Activities button in the “Today’s tasks” section of the home page. In addition, similar to the pattern observed for resources, we observed that, on 68.6% (48/70) of days when an activity was started by a participant, the participant started only 1 activity.

Some participants identified the Activities feature as their favorite and talked about the knowledge they gained from quizzes. They especially appreciated that, even if they gave an incorrect answer at first, the quiz would eventually provide an explanation of the correct answer. One participant noted, “I like the fact that when you give an answer to a quiz, it tells you whether the question is right or not” (18-year-old male participant; time spent=16.8 minutes; SUS score=87.5). This participant also appreciated the variety of activities, enjoying that “they are not only focusing on HIV, but they touch on other topics as well.”

Another participant who named the Activities feature as their favorite sought out quizzes “because I wanted to challenge my brain” and mentioned completing the activities together with their sister. When asked later by the interviewer about their motivation to use the app, the participant described how MASI helped improve their mental health, as seen in the dialogue in Textbox 1.

**Dialogue between an interviewer and a 19-year-old female participant (time spent in the app=50.8 minutes; System Usability Scale score=75).**

Interviewer: What made you use the app on the days that you did?

Participant: It helped me, especially with my mental health so I could keep my mind busy.

Interviewer: I see. That is very nice to hear. Especially that the app helped you with your mental health. Can you expand more on your mental health?

Participant: Most of the time, I would get bored and think about negative things. So, it helped me to just forget about those negative things and keep my mind busy.

#### Ask the Expert

Beta-testing participants collectively spent 13.3 minutes in the MASI Ask the Expert feature. Through this feature, 2 participants posted a question to our doctor, and 2 additional participants “liked” or “favorited” a question asked by someone else. Most of the remaining participants viewed or read content in this section, although 3 participants did not enter this part of the app at all. The participants who posted a question reported that the experience was “really helpful” because their specific questions were “answered explicitly,” as noted by 1 participant:

I was happy because I got more information, and I now know what exactly I need to do and the steps to follow to do things correctly.

19-year-old male participant; time spent=30.1 minutes; SUS score=67.5

Most of the participants who did not submit a question to be answered reported that they “read questions and answers in Ask the Expert and found them useful” but “did not post anything because [they] did not have any question to ask,” as 1 participant summarized (18-year-old male participant; time spent=16.8 minutes; SUS score=87.5). Similarly, another participant who did not use the Ask the Expert feature expressed, “I didn't use that one because I did not have any questions. I feel like the questions that I had on the articles and quiz, they were already answered” (19-year-old female participant; time spent=8.5 minutes; SUS score=65). These comments emphasized that lack of active engagement in one feature was not necessarily a shortcoming of MASI, particularly if the participant was engaged in other features of the app.

## Discussion

### Principal Findings

In this beta-testing study with 12 adolescents with HIV in Cape Town, South Africa, we found high usability of MASI, a smartphone app–delivered adherence-supporting intervention. In this section, we discuss the implications of these findings for technological refinements of MASI and delivery of the MASI intervention.

### Implications for Technological Refinements of MASI

Beta testing MASI allowed our team to explore whether and how the app design facilitated navigation between features and encouraged or discouraged engagement and interaction. Questions about participants’ experiences with technical issues or barriers led us to enhance the app to improve flow across app features. For example, edits to the back button programming were made so that participants could return to their previous screen after they clicked on a MASI link. This enhancement proved particularly important as the team added more cross-feature links to connect participants to relevant resources or activities within MASI related to topics they had been reading about on a previous screen.

Another change made to MASI was based on the finding that the congratulatory message displayed upon completion of a resource or activity in the “Today’s tasks” apparently served to limit some participants’ engagement with additional articles or resources. Ahead of the pilot RCT, the app was programmed to continue displaying new activities and resources even after the first task was completed and the red check mark displayed.

An additional adjustment was made to the MASI platform in response to the sensitive post made during this beta-testing study. Having identified the need to monitor and respond to user-generated content, the MASI platform was configured to generate an email to study administrators whenever there is a new Forum post made. The email contains the text of the Forum post to assist the team with assessing and monitoring posts and responding in a timely manner. A feature to flag key words that may be related to standard operating procedures (SOPs; such as “kill,” “die,” or “hurt”) is in development. Beta testing MASI revealed situations that would require a quick response from our team, allowing us to refine the technological platform to facilitate our monitoring and increase our responsiveness.

Finally, our beta-testing study revealed that the paradata were systematically undercounting the duration of time spent in MASI. This analysis resulted in an update to the app that addressed this issue.

### Implications for Delivery of MASI Intervention

The findings of this beta-testing study had implications for the delivery of the MASI intervention. For example, a fuller understanding of barriers to more active levels of user engagement led the research team to create a content library of posts and prompts (often with corresponding images, GIFS, or YouTube video links) to be posted regularly by the study team throughout the subsequent pilot RCT. Based on feedback obtained in this study, these posts were created to offer opportunities for engagement at varying levels, including polls that can be answered by selecting a radio button, questions prompting 1-word responses, and posts that seek open-ended responses to specific questions. The content of these posts includes material discussed in existing Resources or Ask the Expert questions with a discussion question, “just for fun” content, and “badge alerts” that describe the badges participants can earn by interacting with MASI.

Another implication of this study for the delivery of MASI in subsequent phases was the opportunity to develop tailored content based on search terms that users were inputting when browsing the Resources and Activities sections. As a result of this beta-testing study, subsequent implementation of MASI will include weekly monitoring and internal reporting of search terms used or topics discussed in the Forum that reveal gaps in resource and activity content or a desire for additional resources. The team will prioritize the development of new content during the RCT in response to these data. The paradata serve as an important additional means to capture participant feedback in a way that may be less prone to social desirability bias or recall bias.

Analysis of the qualitative data in addition to the app paradata also revealed mental health as an important area of focus. For example, participants emphasized feelings of not being alone by using the app and described benefitting from activities to keep their mind busy. One participant posted in the Forum about suicidal ideation, and others explicitly mentioned mental health when talking about their engagement with MASI. These data highlight the overall mental health needs of this population and suggest MASI has potential to provide support for an often-stigmatized topic that is central to overall well-being. As a result, the team will also prioritize the generation of new content related to mental health and will continue to look for other ways to support this need.

The findings of this study led to the development of expanded procedures for supporting participants in response to concerns about participant well-being. For example, the participant who posted about suicidality in the Forum prompted the team to develop a comprehensive set of SOPs for various scenarios warranting follow-up with a participant, including procedures for when concerns are raised within the MASI app (eg, text alluding to self-harm, abuse, or violence in a Forum post or Ask the Expert question). The team was able to expand on existing SOPs to add procedures for contacting a participant and assessing risk, in addition to responding to the participant on the MASI app. The SOP includes sample responses to Forum posts that can be shared publicly as well as links to in-app resources that participants can access and to local organizations or services that can be found through the app’s Care Locator. The goal of these procedures is to help establish MASI as a safe place in which participants can discuss real issues that they or others may be facing, including topics that may be stigmatizing (such as mental health), and simultaneously ensure participant safety and well-being. In addition to formalizing SOPs for supporting participants, the team (1) revised the language of informed consent forms to clearly outline instances in which the research team may need to break confidentiality in order to comply with local laws (eg, mandatory reporting) and (2) added training to ensure that team members are aware of their role and responsibilities when responding to concerns, as outlined in the SOPs.

Finally, our beta-testing study highlighted the important role played by the MASI peer mentors. MASI peer mentors were a constant presence throughout the beta-testing study period. This was particularly important given the staggered enrollment of participants and relatively short app access, which limited the number of active users at any given time. When a participant posted a question about suicidality, the peer mentors responded with personal reflections and notes of encouragement. In his follow-up interview, the participant noted how helpful the peer mentors’ responses had been. This feedback underscored the importance of peer mentors’ inclusion and activity in the MASI app, particularly as peer mentors can relate to participants as fellow young people who are also living with HIV.

### Conclusions

Initial usability of MASI, a smartphone app–delivered adherence-supporting intervention, was high among adolescents with HIV in Cape Town, South Africa. Thematic analysis of qualitative results revealed generally positive experiences across MASI features and provided opportunities to refine the app and intervention delivery. Systematically analyzing paradata and using the interview findings to qualitatively explore participant experiences allowed us to gain richer insights into patterns of participant engagement and enabled our team to further enhance MASI. The results from this study led to a few technological refinements to improve the user experience. Enhancements were also made to the intervention implementation plan in preparation for a pilot RCT. Lessons learned from the conduct of this beta-testing study may inform the development, implementation, and evaluation of similar app-delivered interventions in the future.

## Abbreviations

ART: antiretroviral therapy
HMP: HealthMpowerment
LGBTQ: lesbian, gay, bisexual, transgender, and queer
MASI: Masakhane Siphucule Impilo Yethu
RCT: randomized controlled trial
SOP: standard operating procedure
SUS: System Usability Scale
